# A comparison of types and thicknesses of adhesive felt padding in the reduction of peak plantar pressure of the foot: a case report

**DOI:** 10.1186/s13256-015-0675-8

**Published:** 2015-09-24

**Authors:** Michael J. Curran, Connor Ratcliffe, Jackie Campbell

**Affiliations:** School of Health, The University of Northampton, Boughton Green Road, Northampton, NN2 7AL UK

## Abstract

**Introduction:**

This case report will have implications for any area of medicine that aims to redistribute plantar pressure away from a particular area of the foot. This could be for example in the short-term care of people with diabetes, people who have insensate feet and people with poor blood supply to the foot coupled with plantar ulceration. The aim of the study was to investigate which type and thickness of Hapla felt padding is the most effective at redistributing plantar pressure of the foot. This case report is the first of its kind.

**Case presentation:**

The participant was a healthy 50-year-old white man with a high peak plantar pressure over the second metatarsal head of both feet; he required removal of a plantar callus on a periodic basis.

**Conclusions:**

The reader should note that different types of Hapla felt padding provide different forms of redistribution of plantar pressure on the foot. In the clinic it may be useful to measure peak plantar pressure using F-Scan before deciding on the most appropriate type of felt padding.

## Introduction

A healthy 50-year-old white man presented with a long-standing painful plantar callus over the second metatarsal head that required podiatry treatment to remove the callus. After removal of the callus the area was often painful and it required the use of felt padding to offload plantar pressure before orthoses were prescribed for long-term treatment of the condition.

Felt padding has been used for a long time as a material for redistribution of foot pressure from various parts of the plantar surface of the foot, although there is little published research on the effect of felt on plantar pressure. According to Nube *et al*. [1], the purpose of felt padding is to deflect pressure away from a particular area of the foot. They demonstrated that plantar pressure can be reduced in people with diabetes by the application of felt padding that is adhered to the foot. They argued that foot ulceration in diabetes is precipitated and perpetuated by many factors, chiefly peripheral neuropathy and biomechanical abnormalities but offloading of plantar pressure is a key element in the management of diabetes-related neuropathic foot ulcers. The technique principally involves the adherence of felt or felted-foam to the sole of the foot, with an aperture cut into the material which corresponds with the ulcer location. Varying thicknesses of felt and methods of adhesion are used. Holmes and Timmerman [2] used a pedobaragraph to assess the effect of a simple metatarsal pad on pressures transmitted to metatarsal heads by measuring dynamic pressure for 100 participants with and without metatarsal pads. They concluded that, when properly positioned, metatarsal pads can be an inexpensive and effective means of reducing plantar pressure. Hayda *et al*. [3] reported similar findings; they measured plantar pressures within the shoes of 10 volunteers with normal asymptomatic feet. Test conditions included large foam, large felt and small felt situated at three different positions. They concluded that metatarsal pads can effectively decrease plantar pressure in the shoe. A study by Zimny *et al.* [4] evaluated the effects of felted foam on wound healing in diabetes-related foot ulcers compared with a standard method of plantar pressure relief. All patients received identical standard ulcer wound care including debridement and daily careful monitoring of the ulcer. In a randomized trial, the patients received pressure relief in the ulcerated area either with felted foam dressing or with a pressure-relief half-shoe (Thanner, Hoechstaedt, Germany). Zimny *et al.* [4] demonstrated that a dressing of 0.635cm thick rubber foam combined with a 0.158cm layer of felt appeared to be as effective as conventional plantar ulcer treatment using the pressure-relief half-shoe. They postulated that it may be a useful alternative in treating neuropathic foot ulceration, especially in patients who are unable to avoid weight-bearing. Paton *et al*. [5] studied the effect of 7mm semi-compressed felt plantar cover padding, with a U shape cutout to the second metatarsal head, on forefoot peak pressure (PP) and forefoot pressure-time integral (PTI). Both feet of 10 healthy individuals were studied. A F-scan in-shoe pressure analysis system compared the dynamic measurements for each individual, with and without felt plantar cover padding. Related sample *t*-tests were conducted to analyze significant differences between test conditions. The PP and PTI at the center of the 'U' decreased by a mean of 25% and 29% respectively. The PP and PTI at the periphery of the 'U' increased by a mean of 44% and 58%, 24.178kPa/second. Exactly what is determined as high peak plantar pressure in causing foot ulcers is debatable but Armstrong *et al*. [6] found that peak plantar pressure was, as expected, significantly higher for patients with ulcers compared to controls: 83.1 +/− 24.7 N/cm^2^ (range, 10 to 125) versus 62.7 +/− 24.4 N/cm^2^ (range, 7.3 to 113), *p* < .001. The ulcer group was clearly skewed toward a higher prevalence of elevated peak plantar forefoot pressure compared with the control group. They concluded that, while there is no optimal cut-point for clearly screening patients for risk of foot ulceration, the higher the PP, the higher the commensurate risk. It can therefore be deduced from the literature that felt padding can play an important role in the management of patients’ foot health but more research is required in this area as there is a variety of types and thicknesses of felt available.

## Case presentation

A 50-year-old healthy white man with a body mass of 108kg had repeated calluses form underneath the second metatarsal head. He had the callus removed every 6 weeks by a podiatrist and then had some padding applied to alleviate the pain. The aim of this case study was to investigate which combination of adhesive felt padding and thickness of padding is the most effective at redistributing plantar pressure of the foot compared to a control of no felt padding.

In order to measure the amount of plantar pressure present on the second metatarsal head F-Scan sensors were fastened to the inside floor of his shoes using double-sided tape to avoid sensor movement and reduce likelihood of crinkling. The shoes were of a flat slip-on type. The sensors were ‘conditioned’ prior to calibration as recommended by the F-Scan user manual. (This consisted of wearing in the shoe for 20 steps or so, allowing him to become accustomed to the sensors and the sensors to become accustomed to temperature within the shoe.) He wore a standardized thin pair of socks so as to avoid any additional pressure redistribution. The F-Scan equipment was calibrated (using the participant’s previously recorded mass) using the point calibration procedure as recommended in the F-Scan user manual. Calibration was performed at the beginning and end of data collection to ensure the sensor was performing consistently throughout. He demonstrated a high peak plantar pressure over the second metatarsal head of both feet. Five different types of felt that could be adhered to the plantar surface of the foot were obtained (see Table 1).

**Table 1:** Types of felt used and thickness

Type of padding felt	Thickness of the felt
Hapla Soft felt	5mm
Hapla Soft felt	7mm
Hapla Mixture	5mm
Hapla Gold	7mm
Hapla Foam-O-Felt	7mm

Not all thicknesses were available for all types of felt. A cardboard template was used to cutout the padding felt to ensure the same amount of felt was used and the design of the pad remained constant.

**Fig. 1:**
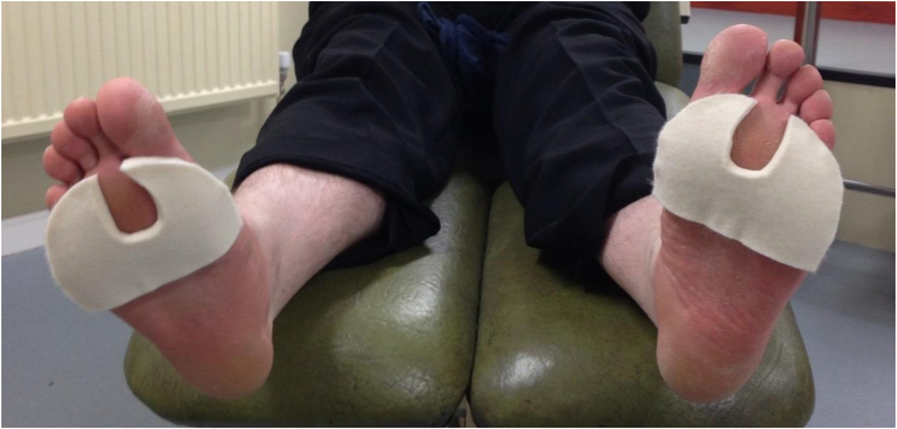
Plantar cover pad with U to second metatarsal head

The adhesive felt padding materials were stuck to the plantar surface of both his feet using the adhesive sticky back integral to the felt pad (see Fig. 1). Readings were obtained from a cycle of gait lasting 20 seconds, which resulted in 15 complete gait cycles per foot. The procedure was then repeated five times for each felt; that is, 75 readings per foot for each felt. A total of 450 readings were completed per foot including a control. He completed several gait cycles whilst wearing the F-Scan sensors to monitor plantar pressure. He was in control of triggering the start of the recording, allowing him to achieve comfort and normality of gait before recording began. A treadmill was used to find a walking pace that he found comfortable; this pace (3.6km/hour) was then used to keep speed of gait consistent. The readings which comprised the final data were taken when he was walking on the treadmill. The padding was then removed from his foot after five runs and another piece of material applied and he repeated the procedure until all five of the materials had been used and the results were recorded before they were entered into SPSS version 20.

Readings were also taken of plantar pressure across his metatarsal head area with no padding material to act as a control. Sensors were used for a maximum of 10 runs to prevent the possibility of sensor degradation. Calibration was performed each time the sensors were changed. The F-scan software was then used to view the entire foot strike at one time, as a ‘stance’; this groups one gait cycle (heel strike through to toe off) into one picture. It was decided to record the PP across his whole metatarsal head area on the F-Scan software, the software still locates the point of highest PP, but this point could be on any of his metatarsal heads. The alternative would be to take readings for each individual metatarsal head; however, it is difficult to clearly, consistently and accurately define each individual metatarsal head using the F-scan software.

The research participant exhibited overloading on the second metatarsal head and the felt padding was designed to reduce the pressure under that point. However, if the off-loading then shifted PP to another metatarsal head there would be a risk of this new point of PP being overloaded. So, for example, a reduction of pressure under the second metatarsal head from 500 to 250kPa would be useful but if pressure under the third metatarsal head is increased to a peak of 550kPa then this may produce unwanted problems, so changes in pressure across the whole metatarsal head region were considered.

The metatarsal head region was defined using the F-Scan software and the researchers’ knowledge of the foot. By using the stance function in the F-Scan software, a graphical representation of loading across the foot for a full gait cycle could be seen. With a basic knowledge of the anatomy of the foot the metatarsal head region can then be visually defined by using the add/edit box feature within the software. This allows PP readings to be obtained solely from the defined area.

In addition, a full assessment of the peak plantar pressures at and on the periphery of the cutout on the felt padding was undertaken. This was necessary to establish whether the cutout increased PPs in the periphery. To this end, we ran through one cycle of 7mm felt (14 data points). We used a box across the whole metatarsal area and boxed each periphery of the cutout. No increase in peak plantar pressure at the periphery of the cutout was found.

### Statistical analysis

**Fig. 2:**
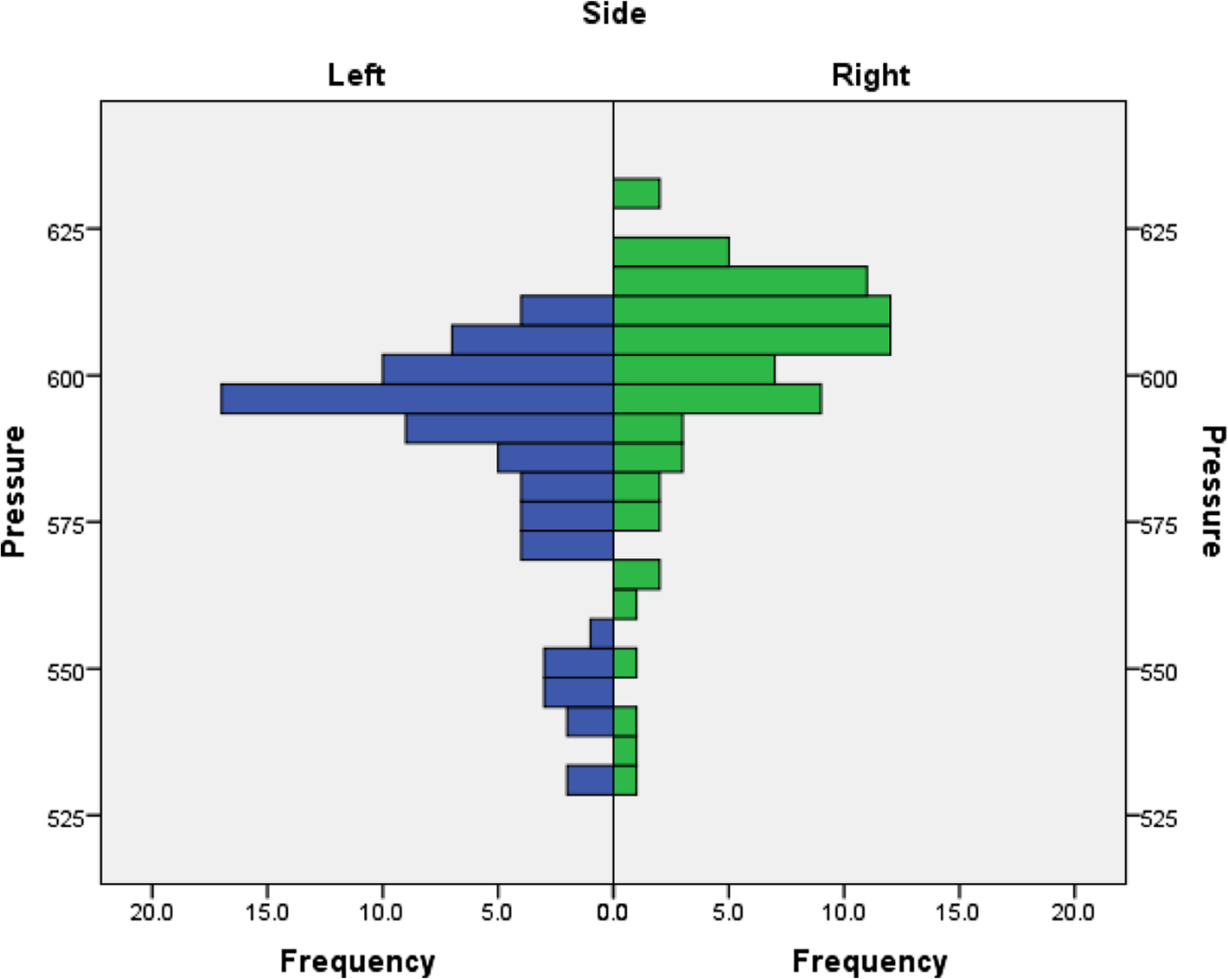
Distribution of peak pressure for left and right feet under no felt (control) condition (75 repetitions)

This is a single participant study with intra-participant replication. The mean PP (with no felt) for the participant’s left foot was 586.68kPa (*n*=75) with a standard deviation of 20.13kPa and for the right foot the mean was 600.01kPa (*n*=75) with a standard deviation of 20.03kPa. This indicates that the variance is approximately the same between the two feet but the PP under the right metatarsal heads is greater than that on the left (see Fig. 2).

The results for this study are presented graphically for visual analysis. The results for each felt (and control) were obtained from 75 repetitions. Because the results for the participant’s left and right foot were different, they are presented separately.

**Fig. 3:**
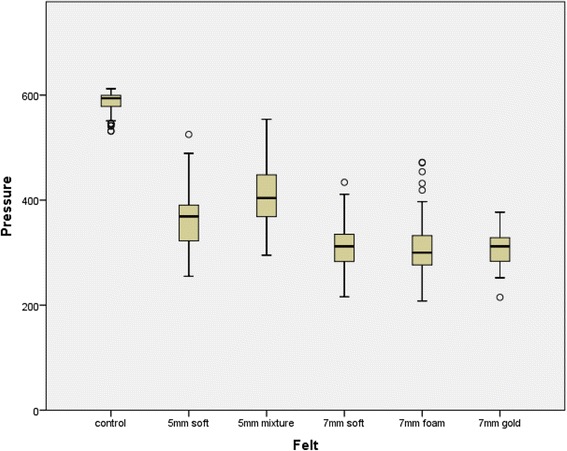
Box plot of the peak pressures under the metatarsal heads for each felt and without felt (control) for the left foot

For his left foot, the control condition (no felt) produced the highest pressures, with 5mm Mixture felt producing an approximately 30% mean pressure reduction. Soft felt of 5mm produced a further slight pressure reduction. The three 7mm felts (Soft, Foam-O-Felt and Gold felt) gave the best pressure reduction (see Fig. 3).

**Fig. 4:**
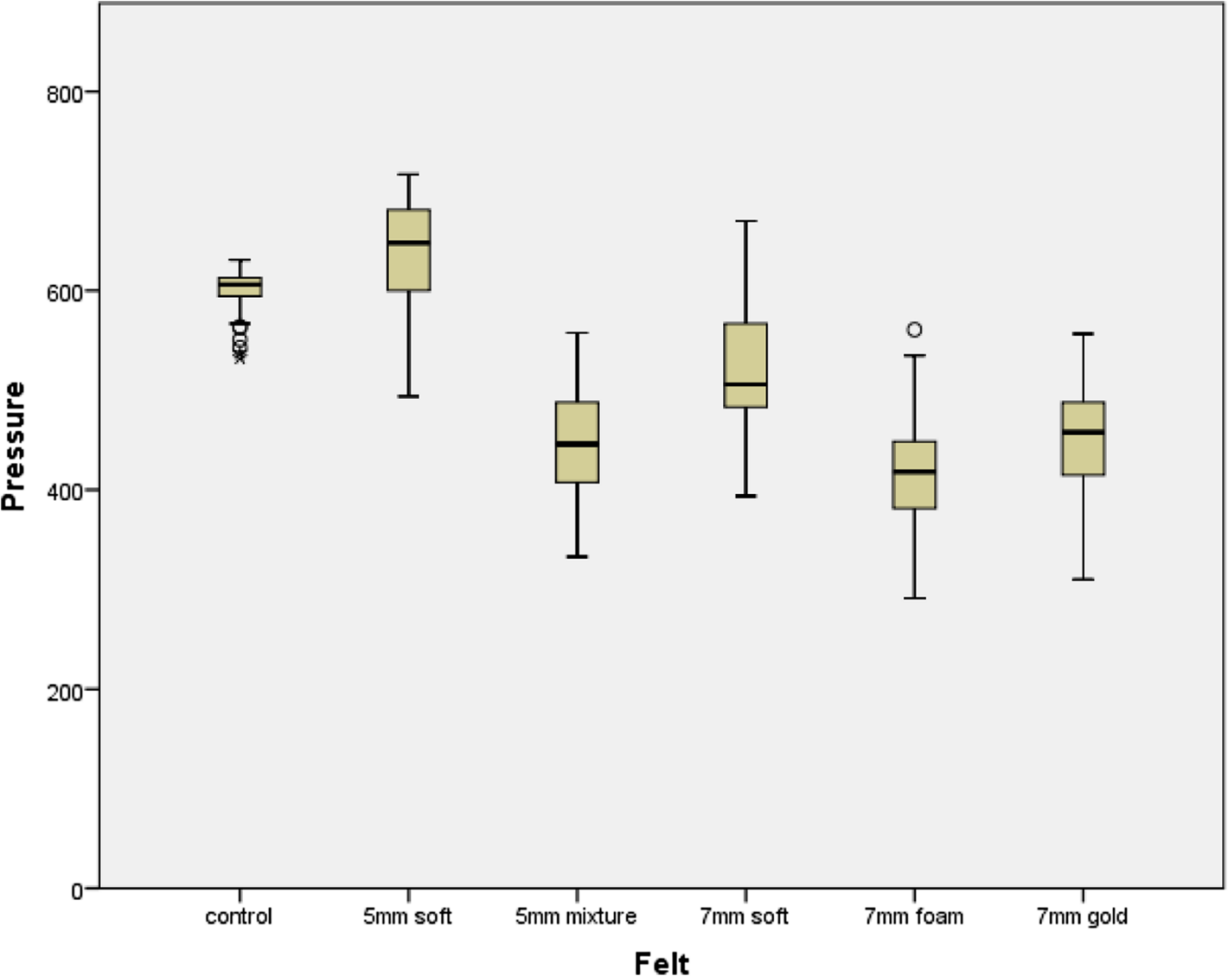
Box plot of the peak pressures under the metatarsal heads for each felt and without felt (control) for the right foot

For his right foot, the pressure reduction was generally lower than for his left and the performance of the different felts was different to that seen previously. Foam-O-Felt of 7mm gave the best pressure reduction, being slightly better than both 5mm Mixture felt and 7mm Gold felt. Soft felt of 7mm also shows a pressure reduction compared to the control. Of interest, the 5mm Soft felt produced a higher peak metatarsal head pressure than using no felt (see Fig. 4). The descriptive statistics for each foot and each felt are shown in Table 2.

**Table 2:** Peak pressure descriptive statistics

Felt	Peak pressure (kPa) under metatarsal heads			
	Left foot		Left foot	
	Mean (standard deviation)	[Minimum, maximum]	Mean (standard deviation)	[Minimum, maximum]
Control	586.68 (20.13)	[531, 612]	600.01 (20.03)	[533, 631]
5mm soft	362.91 (50.55)	[255, 525]	638.96 (50.61)	[494, 717]
5mm mixture	409.49 (56.71)	[295, 554]	446.525 (35.30)	[333, 558]
7mm soft	313.32 (42.74)	[216, 434]	520.41 (56.13)	[394, 670]
7mm foam	309.45 (55.72)	[208, 472]	418.35 (58.84)	[291, 561]
7mm gold	306.73 (32.35)	[215, 377]	449.11 (53.52)	[310, 557]

## Discussion

The aim of the study was to investigate which felt padding would be the most effective at redistributing plantar pressure of the foot compared to a control of no felt padding. Previous authors [1–3] found that felt padding was useful in the deflection of plantar pressure and in the prevention of tissue damage. This case study demonstrates that 7mm felt padding produces greater pressure reduction than the 5mm felt padding. However, there were laterality differences in the redistribution of plantar pressure by the felt padding. For the left foot, where the peak plantar pressure for the control is lower than the right foot, 7mm Gold felt padding, 7mm Foam-O-Felt padding and 7mm Soft felt padding produced the greatest pressure reduction and their effects were similar. For the right foot, where the pressure was significantly higher than the left foot, 7mm Foam-O-Felt padding gave the best pressure reduction, being slightly better than both 5mm Mixture felt and 7mm Gold felt which were statistically indistinguishable. Soft felt of 7mm also gave a statistically significant pressure reduction compared to the control. There was one anomaly in that there was an increase in pressure after using 5mm Soft felt on the right foot. A possible explanation is that for this participant this form of felt may not be able to redistribute plantar pressure as the thickness may decrease immediately.

Two authors have stated that the application of appropriate padding material to the foot is useful in first-line treatments of ulcers [4–6]. To this end, this study has provided an insight into which specific types of felt and which thickness of felt provides for optimum redistribution of plantar pressure. It may be useful to measure peak plantar pressure before deciding which type of felt padding to use and this may be very useful when using felt padding for specific patient groups where redistribution of pressure is critical, for example where a patient has diabetes and a plantar ulcer. The study design has the disadvantages of results from only one participant albeit with a significant higher peak plantar pressure under one foot (right) than the other foot. However, it had the advantage of holding other variables constant, for example body mass, and there were no anatomical variations or gait idiosyncrasies between different participants. It would be interesting in further studies to see what effect parameters such as foot type, variations in footwear and increasing gait velocities would have on the results and to what extent the natural variability between individuals affects the effectiveness of different felts. Paton *et al*. [5] demonstrated that cutouts in felt padding can increase pressures at the periphery of the cutout and this may be detrimental to a patient with for example poor tissue viability. Our study shows that the PP with padding, even at the edge of the cutout, is lower than the PP without padding over the second metatarsal head. Padding reduced PP compared to the control and this included the periphery of the cutout as well as under all other metatarsal heads. This takes into account any undesirable shifting of high pressure from the padded area to the other metatarsal heads through, for instance, changes in gait resulting from the addition of the padding. The present study was not a longitudinal study and no information is available at present to suggest how long the felt padding will be effective in redistributing pressure neither does it indicate when the padding should be changed. In this study peak plantar pressure was measured immediately after its application but it would be interesting to find out what happens after a longer period of time has elapsed, for example 24 to 48 hours later. This could have some influence in the offloading effect especially in people with poor tissue viability.

## Conclusions

Felt padding of 7mm produces a greater reduction in force than 5mm felt padding. It may be worth measuring peak plantar pressure using F-Scan as felt padding may behave differently with different levels of peak plantar pressure. Where peak plantar pressure is high 7mm Foam-O-Felt is the best choice of felt padding. This study may have implications for any area of medicine that redistributes peak plantar pressure. This may be particularly important in the short-term management of people with diabetes or those who have insensate feet and plantar ulceration.

## Consent

Written informed consent was obtained from the patient for publication of this case report and accompanying images. A copy of the written consent is available for review by the Editor-in-Chief of this journal.
